# Lateral flow urine lipoarabinomannan assay for tuberculosis diagnosis in people living with HIV: a step in the right direction, but we need more

**DOI:** 10.36416/1806-3756/e20250329

**Published:** 2025-09-22

**Authors:** Ana Paula Santos, Fernanda Carvalho Queiroz Mello

**Affiliations:** 1. Hospital Universitário Pedro Ernesto, Universidade do Estado do Rio de Janeiro, Rio de Janeiro (RJ) Brasil.; 2. Instituto de Doenças do Tórax, Universidade Federal do Rio de Janeiro, Rio de Janeiro (RJ) Brasil.

Rapid diagnosis of tuberculosis is one of the most important pillars of the WHO End TB Strategy.^1^ This is especially crucial for paucibacillary forms-including extrapulmonary tuberculosis, tuberculosis in children, and in people living with HIV (PLHIV)-which remain major challenges. 

Since 2019, the WHO has recommended the lateral flow urine lipoarabinomannan assay (LF-LAM) to assist in diagnosing active tuberculosis in HIV-positive inpatients and outpatients who meet specific criteria, most of which are related to disease severity and the degree of immunosuppression as measured by CD4 cell counts.^2^ Following these recommendations, the Brazilian National Ministry of Health incorporated LF-LAM into the public health system. PLHIV presenting with clinical manifestations of pulmonary or extrapulmonary tuberculosis (regardless of CD4 cell counts) and those who are severely ill should undergo LF-LAM. Asymptomatic patients should be tested on the basis of their CD4 cell counts, i.e., < 200 cells/mm^3^ for inpatients and < 100 cells/mm^3^ for outpatients.^3^

Lipoarabinomannan is the most extensively studied immunomodulatory lipid in the mycobacterial cell wall and contributes to the pathogenicity of virulent mycobacteria by reducing the host protective response and evading immunity.^4^ Following the degradation of *Mycobacterium tuberculosis*, the remaining free lipoarabinomannan in the bloodstream is filtered through the glomerular basement membrane into the urine. Using an immunochromatographic test, antibodies against lipoarabinomannan on a strip react with this antigen, producing a colored line that is interpreted as reactive. 

In this issue of the *Jornal Brasileiro de Pneumologia*, Pereira et al.^5^ present the results of LF-LAM (DETERMINE™ TB LAM Ag test; Abott Laboratories, Abott Park, IL, USA) for tuberculosis diagnosis in PLHIV in southern Brazil. In their study,^5^ LF-LAM detected an additional 8.6% of tuberculosis cases in comparison with rapid molecular tests on sputum. LF-LAM-positive patients were younger and had lower CD4 counts, whereas smoking was more common among LF-LAM-negative patients.^5^

The literature, however, shows inconsistent results regarding the performance of LF-LAM for tuberculosis diagnosis. One systematic review and meta-analysis showed a wide range of sensitivities (from 8% to 80%), whereas specificity ranged from 88% to 99%.^6^ More recently, a diagnostic yield of 41% was reported for LF-LAM, being 61% for rapid molecular tests and 32% for sputum smear microscopy.^7^

Despite its suboptimal sensitivity for routine clinical use, LF-LAM has several characteristics that make it attractive for tuberculosis diagnosis. The easier availability of urine in comparison with that of sputum or other biological specimens, the reasonable cost of the test, and its use as a point-of-care test are some of the advantages. These characteristics have led to a reduction in eight-week mortality in a study conducted in Africa,^8^ where patients were often severely ill, had advanced immunosuppression, were unable to produce sputum, and were living in settings with scarce diagnostic resources. In such patients, the speed of tuberculosis diagnosis can be the difference between a favorable and an unfavorable outcome. 

Although LF-LAM shows benefits in reducing mortality among PLHIV, the occurrence of false-positive and invalid results should be taken into account by clinicians. In a recent study,^9^ approximately 20% of LF-LAM-positive results were attributed to nontuberculous mycobacterial disease, nocardiosis, and cryptococcosis-conditions that must be included in the differential diagnosis of PLHIV. Another 7% of cases showed invalid results in patients with chronic kidney disease.^9^

Importantly, molecular and/or microbiological tuberculosis investigations must continue in order to identify the mycobacteria and determine *M*. *tuberculosis* drug susceptibility, given that LF-LAM has shown comparable sensitivity for diagnosing tuberculosis and nontuberculous mycobacterial disease,^10^ and cannot detect drug-resistant tuberculosis. 

The first commercial LF-LAM was the DETERMINE™ TB LAM Ag test (Alere, Waltham, MA, USA). The next-generation LF-LAM, SILVAMP TB LAM (Fujifilm, Tokyo, Japan) promises increased sensitivity as a result of its ability to detect lower lipoarabinomannan concentrations. Despite this improvement, variability in accuracy between SILVAMP TB LAM lot numbers, as identified by Huerga et al.,^11^ has reduced its reliability. For this reason, the next-generation LF-LAM has yet to be endorsed for clinical use by the WHO. 

Research is ongoing to develop and evaluate other lipoarabinomannan-based tests for tuberculosis diagnosis in PLHIV and non-HIV patients, as well as using specimens other than urine. Another potential application that is currently being studied is the use of lipoarabinomannan tests to monitor tuberculosis patients during treatment. The electrochemiluminescence lipoarabinomannan research assay has been used in order to measure this antigen in the urine of HIV-negative individuals with pulmonary tuberculosis and to monitor antituberculosis treatment.^12^ The results suggest that there is a strong possibility of meeting the WHO target product profile for tuberculosis diagnosis in the near future. 

Although LF-LAM can be very helpful in PLHIV, particularly those who are hospitalized, as shown by Pereira et al,^5^ it still requires careful interpretation in a clinical context. 

## References

[1] World Health Organization [homepage on the Internet] The end TB strategy.

[2] World Health Organization (2019). Lateral flow urine lipoarabinomannan assay (LF-LAM) for the diagnosis of active tuberculosis in people living with HIV, 2019 Update.

[3] Ministério da Saúde. Secretaria de Vigilância em Saúde e Ambiente. Departamento de HIV/AIDS, Tuberculose, Hepatites Virais e Infecções Sexualmente Transmissíveis (2023). Coordenação-Geral de Vigilância da Tuberculose, Micoses Endêmicas e Micobactérias não Tuberculosas. Nota Informativa Nº 6/2023-CGTM/DVIAHV/SVSA/MS. Recomendações para uso do teste rápido LF-LAM para diagnóstico de tuberculose em pessoas vivendo com HIV/aids.

[4] Sarkar P, Biswas D, Sindhwani G, Rawat J, Kotwal A, Kakati B (2014). Application of lipoarabinomannan antigen in tuberculosis diagnostics current evidence. Postgrad Med J.

[5] Pereira GR, Barbosa MS, Secchi C, Passos MP, Santos AK, Braga RSL (2025). Lateral flow urine lipoarabinomannan assay for tuberculosis diagnosis in HIV-positive inpatients and outpatients. J Bras Pneumol.

[6] Minion J, Leung E, Talbot E, Dheda K, Pai M, Menzies D (2011). Diagnosing tuberculosis with urine lipoarabinomannan systematic review and meta-analysis. Eur Respir J.

[7] Broger T, Koeppel L, Huerga H, Miller P, Gupta-Wright A, Blanc FX (2023). Diagnostic yield of urine lipoarabinomannan and sputum tuberculosis tests in people living with HIV a systematic review and meta-analysis of individual participant data. Lancet Glob Health.

[8] Peter JG, Zijenah LS, Chanda D, Clowes P, Lesosky M, Gina P (2016). Effect on mortality of point-of-care, urine-based lipoarabinomannan testing to guide tuberculosis treatment initiation in HIV-positive hospital inpatients a pragmatic, parallel-group, multicountry, open-label, randomised controlled trial. Lancet.

[9] Sharma S, Kumar D (2025). Unravelling the Enigma Evaluating False Positive and Invalid Results in Urinary TB LAM Test among HIV Patients with Opportunistic Infections. Open Forum Infect Dis.

[10] Liu D, Gu L, Zhang R, Liu L, Shen Y, Shao Y (2022). Utility of urine lipoarabinomannan (LAM) in diagnosing mycobacteria infection among hospitalized HIV-positive patients. Int J Infect Dis.

[11] Huerga H, Bastard M, Lubega AV, Akinyi M, Antabak NT, Ohler L (2023). Novel FujiLAM assay to detect tuberculosis in HIV-positive ambulatory patients in four African countries a diagnostic accuracy study. Lancet Glob Health.

[12] Budde K, Lange C, Reimann M, Zielinski N, Meiwes L, Köhler N (2025). A novel method for detecting Lipoarabinomannan in urine with the promise of meeting the WHO target product profile for the diagnosis of tuberculosis. Tuberculosis (Edinb).

